# How Probiotics Affect the Microbiota

**DOI:** 10.3389/fcimb.2019.00454

**Published:** 2020-01-15

**Authors:** Grégoire Wieërs, Leila Belkhir, Raphaël Enaud, Sophie Leclercq, Jean-Michel Philippart de Foy, Isabelle Dequenne, Philippe de Timary, Patrice D. Cani

**Affiliations:** ^1^Service de Médecine Interne Générale, Clinique Saint Pierre, Ottignies, Belgium; ^2^Service de Médecine Interne et Maladies Infectieuses, Cliniques Universitaires Saint Luc, UCLouvain, Université Catholique de Louvain, Brussels, Belgium; ^3^CHU Bordeaux, CRCM Pédiatrique, CIC 1401, Université de Bordeaux, INSERM, CRCTB, U1045, CHU Bordeaux, Bordeaux, France; ^4^Institute of Neuroscience and Louvain Drug Research Institute, UCLouvain, Université Catholique de Louvain, Brussels, Belgium; ^5^Société Scientifique de Médecine Générale, Cellule Nutrition, Brussels, Belgium; ^6^Société Royale Belge de Dermatologie, Brussels, Belgium; ^7^Service de Psychiatrie, Cliniques Universitaires Saint Luc, UCLouvain, Université Catholique de Louvain, Brussels, Belgium; ^8^Walloon Excellence in Life Sciences and BIOtechnology, Metabolism and Nutrition Research Group, Louvain Drug Research Institute, UCLouvain, Université Catholique de Louvain, Brussels, Belgium

**Keywords:** probiotic, microbiota, metabolism, skin, psychiatry, drug interaction, clinics

## Abstract

Probiotics have been used to treat a variety of diseases for decades; however, what is the rationale for their application? Such a treatment was first proposed in the early nineteenth century based on observations of decreased bifidobacterial populations in children suffering from diarrhea, suggesting that oral intake of bifidobacteria could replete this subpopulation of the microbiota and improve health. Since then, studies have shown modifications in the gut or skin microbiota in the course of a variety of diseases and suggested positive effects of certain probiotics. Most studies failed to report any impact on the microbiota. The impact of probiotics as well as of bacteria colonizing food does not reside in their ability to graft in the microbiota but rather in sharing genes and metabolites, supporting challenged microbiota, and directly influencing epithelial and immune cells. Such observations argue that probiotics could be associated with conventional drugs for insulin resistance, infectious diseases, inflammatory diseases, and psychiatric disorders and could also interfere with drug metabolism. Nevertheless, in the context of a plethora of probiotic strains and associations produced in conditions that do not allow direct comparisons, it remains difficult to know whether a patient would benefit from taking a particular probiotic. In other words, although several mechanisms are observed when studying a single probiotic strain, not all individual strains are expected to share the same effects. To clarify the role of probiotics in the clinic, we explored the relation between probiotics and the gut and skin microbiota.

## A Short History of Probiotics

The first report of voluntary modification of the gut microbiota was described in ancient China with the use of human feces to treat infections or food poisoning. Indeed, the intervention to modify the microbiota via the use of fecal material has been described for more than 500 years, but the use of specific strains of bacteria to obtain a specific clinical impact has been of interest for only 50 years. In fact, the first definition of probiotics was produced in 1965 by Lilly and Stillwell and was restricted to substances produced by bacteria that promote the growth of other bacteria (Lilly and Stillwell, 1965).

In 1989, the notion of a living microbial complement appeared, although this definition was still linked only to nutritional health (Fuller, 1989; Huis in't Veld et al., 1994). The last and current definition considers probiotics to be living microorganisms that must be ingested in a sufficient amount to have a positive effect on health that is not limited to the nutritional effects (Guarner and Schaafsma, 1998; Hill et al., 2014). All three definitions provide insight into how probiotics can impact health: by impacting the resident microbiota, intestinal epithelium cells and, globally, the immune system.

The first available probiotics contained only one species of microorganisms, mainly those from the *Saccharomyces* or *Lactobacillus* genera. Subsequent trials were collected in meta-analyses that showed an advantage conferred by the use of such probiotics on the prevention of infectious diarrhea and post antibiotic diarrhea as colitis due to *Clostridium difficile* (Goldenberg et al., 2017).

Subsequent forms of probiotics contained a larger variety and number of microorganisms, ranging from 10^8^ to more than 10^10^ organisms. Most strains of probiotics were developed for their capacity to resist low gastric pH, giving rise to a plethora of variants with unknown physiological properties. Such a great variety of microorganism associations makes comparisons difficult, giving an impression of drug class effects and leading to inadequate prescriptions of probiotics, while the lack of independent studies obscures the probable physiological effects associated with each of these strains (West et al., 2014; de Simone, 2019; Ohkusa et al., 2019). The prebiotic role of cell component of dead probiotic bacteria is another confusing consideration that is not enough studied. *Next-generation probiotics* have better defined properties and clinical indications (Satokari, 2019). However, in addition to the use of specific probiotics, it has also been shown that the coevolution of humans with specific bacteria has led to beneficial effects. One of the best examples is the link between the bacteria *Bacteroides plebeius* in the Japanese population. Indeed, bacteria colonizing foods challenge the microbiota to adapt by lateral gene transfer, which is exactly what *B. plebeius*, a commensal of the microbiota of Japanese people, is showing. By inheriting enzymes produced by the marine bacteria *Zobellia galactanivorans, B. plebeius* in the gut allows the Japanese population to digest the algae polysaccharide porphyrin (Hehemann et al., 2010; Sonnenburg, 2010).

In conclusion, the clinical science of probiotics is still progressing slowly not only because of the evolution of the field and the numerous novel bacteria discovered every year but also because of the lack of well-conducted, independent clinical trials since, given the diversity of probiotic candidates, they are too often considered all equally potent and therefore inadequately investigated.

## Probiotics in the Gut Microbiota Ecosystem

The microbiota organizes as a focal ecosystem and changes from one site to another, especially when comparing microbes living in mucus or attached to the intestinal wall, known as the parietal microbiota, with microbes living in food in transit and stools known as the luminal microbiota (Sonnenburg et al., 2006; Lee et al., 2013; Caballero et al., 2015). Microbiota composition is dynamic and individualized depending on the influence of diet, exposition to ingested probiotic bacteria, environmental conditions of the intestine and other factors associated with the host that will include “transiently” some new strains in the ecosystem (Derrien and van Hylckama Vlieg, 2015; Zhang et al., 2016).

The luminal microbiota is altered during a probiotic treatment, reflecting survival during transit through the digestive tract. The stool microbiota compositions before and after probiotic courses ranging from 6 weeks to 6 months with daily > 10^9^ CFU *Lactobacillus rhamnosus* DR20, LGG, NCFM or LA-5 or *Bifidobacterium* BB-12, W23, W52, W58, or Bi-07 were evaluated by culture, 16S PCR or enzymatic tests on healthy adult or infant stools. These probiotic bacteria were retrieved in >90% of the subjects' stools without affecting microbiota composition or diversity when compared to those of the placebo group; interestingly, *Lactobacillus* and *Bifidobacterium* strains corresponding to probiotic bacteria were naturally retrieved in 2–31% of the untreated subjects (Larsen et al., 2011; Dotterud et al., 2015; Rutten et al., 2015; Avershina et al., 2016; Laursen et al., 2017). In another trial, *L. rhamnosus DR20* became dominant inside the population of lactobacilli in 6/10 subjects, which represented 3.10^5^ CFU per gram of stool, but 2 months after treatment cessation, only 1/10 subjects was still colonized (Tannock et al., 2000). Ten days after treatment with *Lactobacillus* GG, the probiotic was retrieved in the stools of all subjects, as the dominant lactobacilli and aerobic and anaerobic bacteria were more numerous in stool samples (Alander et al., 1997). However, is there any clinical implication of survival of the probiotic bacteria in the luminal microbiota? It appears that during a treatment with *L. rhamnosus, Enterococcus* strains were retrieved at an increased frequency and number (Tannock et al., 2000). This alteration could in turn promote or disrupt focal colonies of interacting microorganisms, especially in the context of colonization of multidrug resistant bacteria. Treatment with fermented milk containing probiotics was associated with a gain in microbiota evenness and increased enzymatic pathways implicated in carbohydrate metabolism as well-yogurt intake containing *B. animalis* ssp. lactis CNCM I-2497 increased the level of potential butyrate producing bacteria that will in turn influence systemic metabolism (Johansson et al., 1998; McNulty et al., 2011; Ki Cha et al., 2012; Veiga et al., 2014; Zmora et al., 2018).

Parietal microbiota alteration by probiotics could be even more important for influencing systemic metabolism, such as insulin resistance. Culture of biopsies of the distal colon of subjects treated with 1.2 × 10^11^ *Lactobacillus* GG ATCC 53103 per day for 12 days revealed the presence of 1.6–5 × 10^5^ *Lactobacillus* GG per biopsy in 4/5 subjects, which was 2-fold higher than the number of *Lactobacillus* GG in the proximal colon. Probiotic bacteria remain in the minority when compared to the resident microbiota, and interestingly, the only subject who was not colonized by the probiotic suffered from ulcerative colitis (Alander et al., 1997).

## Probiotics Interaction with Eukaryotic Cells

The bacterial wall or cytosolic molecules can directly interact with immune cells, especially when immune cells and epithelial cells sample the digestive microenvironment or when gut barrier permeability is altered. The first mechanism could lead to immunoregulatory functions, and the second mechanism could lead to endotoxemia associated with insulin resistance, diabetes, and increased cardiovascular morbidity (described later in this review).

Transient probiotic colonization influence on gene expression or metabolic pathways could be more significant than the inclusion of a new strain, as suggested by studies completed in twin pairs concordant for leanness or obesity, showing that the core set of genes and metabolic pathways are better preserved than the bacterial composition of the microbiota (Turnbaugh et al., 2009). van Baarlen et al. investigated the mucosal transcriptome response to three lactobacilli strains and evaluated seven healthy volunteers successively treated with 10^10^ *Lactobacillus acidophilus* Lafti-L10, *Lactobacillus casei* CRL-431, *L. rhamnosus* GG, or placebo. Duodenal biopsies were collected by endoscopy after each cycle of treatment. RNA analyses revealed a strain-specific epithelial response: *L. acidophilus* modulated regulation of the immune response, hormonal regulation of tissue growth and development, and ions homeostasis; *L. casei* modulated proliferation, Th1-Th2 balance and hormonal regulation of blood pressure; and *L. rhamnosus* modulated wound healing, the IFN response and ions homeostasis (van Baarlen et al., 2011). In the case of *Akkermansia muciniphila*, the outer membrane protein Amuc_1100 was shown to directly interact with the TLR-2 receptor, thereby reinforcing the gut barrier, decreasing inflammation and eventually improving health status (Plovier et al., 2017).

## Probiotics and the Gut Barrier Function

Although gastrointestinal cells are continually exposed to microbial antigens and metabolites, we live in perfect symbiosis with these microorganisms. This arrangement is made possible through various elements. Under normal conditions, gut barrier function is highly efficient because of complex multidimensional mechanisms, such as the presence of a mucus layer, tight junction proteins, antimicrobial factors, immunoglobulin A, and sentinels, including intraepithelial lymphocytes and other adaptive immune cells (reviewed in Konig et al., 2016; Wells et al., 2017).

In addition to the conventional immune aspects, the interactions between gut bacteria and the immune system have led to the breakthrough understanding that microbial components or receptors also contribute to the regulation of energy, glucose, and lipid metabolism (Cani et al., 2007; Everard et al., 2014; Duparc et al., 2017) (for review Cani, 2018; Cani et al., 2019). Briefly, in 2007, Cani et al. proposed the concept of metabolic endotoxemia (Cani et al., 2007). Indeed, models of both genetic or diet-induced obesity and diabetes were characterized by an increased level of circulating lipopolysaccharides (LPS) (Cani et al., 2007). Notably, a small increase in blood LPS (i.e., 2- to 4-fold above the basal levels) was found to be a key factor triggering the onset of low-grade inflammation and eventually insulin resistance during obesity and related cardiometabolic disorders. Importantly, this finding was later confirmed in several large human cohorts (Amar et al., 2008; Gummesson et al., 2011; Lassenius et al., 2011; Laugerette et al., 2011; Monte et al., 2011; Pussinen et al., 2011; Horton et al., 2014; Jayashree et al., 2014; Radilla-Vazquez et al., 2016; Gomes et al., 2017). However, the existence of metabolic endotoxemia does not yet prove the causal link between the gut microbiota and the onset of prediabetes.

It was then identified that the major mechanism involved in the development of metabolic endotoxemia was directly connected with an alteration of the gut barrier function and the gut microbiota composition (Cani et al., 2008; Dewulf et al., 2013). In addition to the specific changes in the composition of the gut microbiota, it is proposed that T cells accumulate in the gut of obese subjects consuming high-fat diets, an observation that correlates with morbidity (Monteiro-Sepulveda et al., 2015). In addition, it is suggested that specific immune cells, such as mucosa-associated invariant T cells (MAITs) (i.e., innate-like T cells) exhibiting elevated Th1 and Th17 cytokine production are decreased in obese and type 2 diabetic patients (Magalhaes et al., 2015).

**Figure 1:**
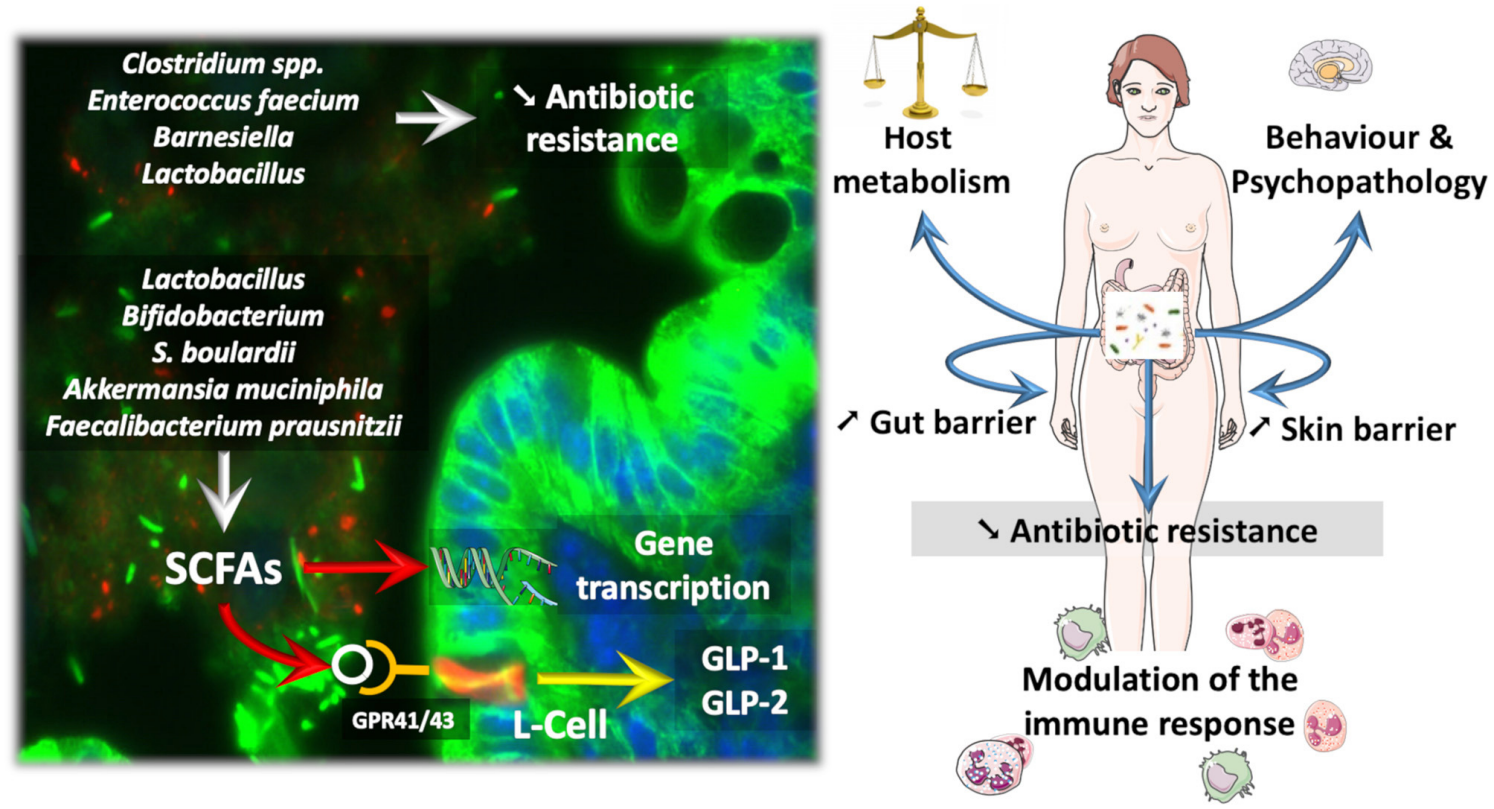
How probiotic influence the microbiota and the course of diseases. Probiotics and next-generation beneficial bacteria influence eukaryotic cells by different mechanisms. For instance, Short Chain Fatty Acids (SCFAs) are able to activate specific G-protein coupled receptors (e.g., GPR41/43) expressed on enteroendocrine L-cells, thereby triggering the secretion of different gut peptides (i.e., GLP-1, GLP-2) involved in the regulation of energy metabolism and gut barrier function. SCFAs can also modulate gene transcription through the inhibition of histone deacetylase activity. Besides SCFAs, some gut microbes dialogue with the host cells through the production of other specific metabolites or cell components. Therefore, such interactions result in a variety of effects on the host ranging from the improvement of behavior in psychopathological conditions (e.g., alcoholism, autism), but also impacts on skin health and host metabolism by the mean of immune interaction and Gut—Brain—Skin axis. Also, bacteria colonizing the normal microbiota as *Barnesiella* have been associated with a reduced susceptibility to gut colonization with Vancomycin resistant *Enterococcus*, whereas, *Lactobacillus* treatment reduced the carriage of multi-drug resistant potential pathogens.

Along these lines, several reports have validated the fact that manipulating the gut microbiota by using probiotics as well as fecal material transplantation may affect host metabolism (Vrieze et al., 2012; Khan et al., 2014; Udayappan et al., 2014; Kootte et al., 2017). The relevant literature focuses largely on various strains, ranging from classical probiotics, such as the bacteria *Lactobacillus* and *Bifidobacterium* or the yeast *Saccharomyces boulardii*, to more recent candidates, such as *A. muciniphila* and *Faecalibacterium prausnitzii*, which are considered next-generation beneficial bacteria (Figure 1) (O'Toole et al., 2017). All these candidates have promoted reinforcement of the gut barrier, reduced inflammation, and eventually improved glucose homeostasis (for review Cani and Van Hul, 2015; Bron et al., 2017; O'Toole et al., 2017; Hiippala et al., 2018).

Several dozens of specific strains of *Lactobacillus* or *Bifidobacterium* have been studied in various protocols and models (for review Cani and Van Hul, 2015; Bron et al., 2017; Borgeraas et al., 2018; Hiippala et al., 2018). The yeast *S. boulardii* is also widely studied in the context of gut barrier dysfunction and inflammation. Moreover, the beneficial effects of this yeast are explained by mostly antimicrobial and antitoxin activities but also by trophic effects on the gut mucosa (McFarland, 2010). More recently, it has been shown that *A. muciniphila* acts as a gatekeeper, consequently improving gut barrier function by restoring mucus layer thickness, tight junction proteins, and production of specific antimicrobial and bioactive lipids with anti-inflammatory properties (Figure 1) (Everard et al., 2013; Plovier et al., 2017; Grander et al., 2018; Hanninen et al., 2018). These activities suggest that *A. muciniphila* interacts with the host mucosal defense by acting on different targets, and today, it is even considered a next-generation beneficial microbe (Cani and de Vos, 2017). For the first time, *A. muciniphila* has been administered to humans. The proof-of-concept study showed that overweight or obese subjects supplemented with *A. muciniphila* for 3 months displayed a lower plasma LPS as well as better insulin sensitivity and lower systemic and liver inflammatory markers than control subjects. Therefore, although not considered a probiotic, *A. muciniphila* is showing promising preliminary data in humans (Depommier et al., 2019). Similar to *A. muciniphila, F. prausnitzii* is another commensal depleted in obese subjects and type 2 diabetic patients and also during inflammatory bowel diseases (Sokol et al., 2008; Thingholm et al., 2019) that displays specific anti-inflammatory properties on the gut (Sokol et al., 2008). Among the different mechanisms involved in reinforcement of the gut barrier, the bacteria and yeast detailed above act via different pathways, including the production of short-chain fatty acids (e.g., butyrate and propionate) that contribute to the regulation of numerous functions ranging from the regulation of gene expression via histone deacetylases to binding to specific G protein-coupled receptors, such as GPR-43 and GPR-41 (Le Poul et al., 2003; Kimura et al., 2014). By activating GPR-41 and GPR-43 expressed on enteroendocrine L-cells, both propionate and butyrate promote the secretion of gut peptides such as glucagon-like peptide-1 and−2 (GLP-1 and GLP-2) involved in the regulation of insulin sensitivity and gut barrier function, respectively (Nohr et al., 2013; for review Rastelli et al., 2019) (Figure 1).

It is worth noting that different strains are not equally potent in terms of their impact on the gut barrier, inflammation, body weight or fat mass, and glucose metabolism. This observation may be explained by the different mechanisms of action described earlier. Hence, it is critical to emphasize the need to consider the metabolic effects of some bacteria as strain specific, which cannot be generalized to all the members of a given genus. In other words, although several mechanisms are observed when studying a single strain, not all individual strains are expected to share the same effects.

## Probiotics in Psychopathology

A large number of human and animal studies support the fact that the gut microbiota plays an important role in cognitive development and function, mood and emotion regulation, and interpersonal interactions and communications, in part through the role of the immune system in neuronal differentiation, axonal development, and synaptic plasticity, serving as a major actor in neuro-immune integration (Figure 1) (Sarkar et al., 2018). Furthermore, impairments in gut microbiota composition have been associated with various psychiatric disorders, such as autism spectrum disorders, major depression, and alcohol-related disorders. For instance, the gut microbiota of depressed patients showed a decrease in richness and diversity associated with an increase in proinflammatory status and cortisol level and alterations in tryptophan metabolism. The transplantation of the microbiota of depressed patients to microbiota-depleted rats induced characteristics of depression at both the behavioral and physiological levels (Kelly et al., 2016). Various pathways have been suggested to explain the relationship between the gut and the brain, including humoral pathways that may transfer inflammatory factors or metabolites, changes in tryptophan metabolism induced by inflammation that may alter neuronal responsiveness, or neural pathways, in particular through vagus nerve activity. This gut-to-brain interaction raises the interesting possibility that the addition of probiotic supplements might serve as an intervention in psychopathology. In 1910, Hubert J. Norman and Georges Porter Philipps had already observed an improvement in depression symptoms after lactic bacilli intake. Since then, numerous efforts have been made to assess the effects of probiotics, using mainly various strains of lactobacilli or *Bacillus infantis*. In animal models, probiotic supplementation was shown to at least partially reverse the behavioral alterations observed in the germ-free model, observed in models of maternal separation-induced depression, or induced by exposure to acute stressors. In human studies, probiotics were shown to improve the inflammatory status of irritable bowel syndrome, to decrease anxiety in chronic fatigue syndrome and psychological distress in healthy volunteers and to modify the treatment of emotional information by the brain, as measured by fMRI scans. The mechanism of action of probiotic supplementation has been shown to not radically alter the gut microbiota composition. Therefore, it is suggested that probiotics target specific interventions. Questions remain, however, regarding the mechanisms by which probiotics might influence behaviors. A gut pathogen, such as *Campylobacter jejuni*, could influence behavior without inducing inflammation. Bravo et al. (2011) showed that administration of *L. rhamnosus* was associated with a decrease in stress-induced anxiety-like behaviors and corticosterone levels and with a regulation of GABA receptor mRNA expression in the brain, effects that were not observed in vagotomized animals, which supports the importance of the vagal nerve in the effects of probiotics (Bravo et al., 2011). However, other studies show that *F. prausnitzii* may exert a direct psychophysical effect by improving gut function and intestinal bowel syndrome (Miquel et al., 2016) by mechanisms described in the gut barrier section of this review.

## Probiotic Eradication of Antibiotic Resistance

Multidrug resistant bacteria, such as vancomycin resistant enterococcus (VRE), carbapenemase-producing enterobacteria (CPE), and extended-spectrum beta-lactamase (ESBL)-carrying strains, represent a major public health issue because they are potential pathogens associated with a high mortality rate (Caballero et al., 2015). Prevention strategies could be based on the use of probiotics to prevent colonization of the colon microbiota (Figure 1).

Transient colonization with multidrug resistant bacteria could result in the transfer of antibiotic resistance genes in commensals or potential pathogens, resulting in the persistence of the resistance gene in the microbiota, which could be responsible for an increased risk of lethal infection due to the delay in introducing an effective antibiotic (Kaushik et al., 2019). Clinical case reports showed that fecal transplantation was able to decolonize the microbiota of ESBL-carrying and naturally resistant bacterial strains (Singh et al., 2014; Crum-Cianflone et al., 2015; Millan et al., 2016). Likewise, the microbiota composition in hospitalized patients was shown to impact the susceptibility to colonization with multidrug resistant bacteria, and the use of probiotic strains such as *L. plantarum* or *L. fermentum* was associated with a reduction in colonization with naturally resistant pathogens, such as *Acinetobacter baumannii, Pseudomonas aeruginosa*, or *Candida albicans* (Singhi and Kumar, 2016; Soltan Dallal et al., 2017). *In vitro*, culture supernatants of *Clostridium butyricum, C. difficile, Clostridium perfringens, Enterococcus faecium*, and *L. plantarum* suppressed the growth and gene resistance transmission of ESBL-carrying bacteria and CPE (Kunishima et al., 2019).

VRE seems less adapted to survival in the gut microbiota and more susceptible to decolonization than other multidrug resistant bacteria. The gut microbiota in patients suffering from hematological malignancies is less frequently colonized with VRE in the presence of *Barnesiella* (Ubeda et al., 2013). Treatment with *Barnesiella* or *Lactobacillus paracasei CNCM I-3689* reduced VRE colonization in a mouse model (Figure 1) (Tannock et al., 2000; Crouzet et al., 2018). In clinics, one case report showed VRE decolonization after a fecal graft for the treatment of *C. difficile* colitis (Stripling et al., 2015).

## Skin Barrier and Probiotics

A large variety of niches host bacteria, such as *Staphylococcus, Corynebacterium* and *Propionibacterium*, which represent 60% of bacterial strains, along with archaea, viruses, fungi, and even mites. *Propionibacterium* dominates oily sites, such as the forehead; *Staphylococcus* prefers moist sites, such as elbow creases and feet; and fungi, primarily of the genus *Malassezia*, live all over the body but are most common in oily areas, such as the face and back (Chen et al., 2018). The microbiota acts in a web-like interaction to suppress virulence-related genes and promote genes associated with commensalism; by producing bioactive molecules, the microbiota influences adnexal development, tumorigenesis, aging, sensory nerve function, and the innate immune system (Figure 1) (reviewed in Belkaid and Tamoutounour, 2016).

As proposed in the epimmunome theory, the barrier status is fundamental for skin defense and immune orientation (Swamy et al., 2010). Skin immune conditions, such as rosacea, acne, and atopy, are associated with skin barrier disruption, and the restoration of the barrier is associated with an improvement in clinical outcomes (Deng et al., 2019). A local application of the probiotics *L. bulgaricus, L. acidophilus*, or *L. plantarum* improves the outcome of acne by reducing skin colonization by *Cutibacterium acnes* (Bowe and Logan, 2011; Muizzuddin et al., 2012).

Soluble proinflammatory molecules, such as substance P, associated with the propagation of skin inflammation, are reduced after local application of *L. paracasei*, and keratinocyte expression of the NF-kB pathway is inhibited after local application of *Streptococcus salivarius* K12 (Cosseau et al., 2008; Gueniche et al., 2010). Likewise, production of the anti-inflammatory molecule IL-10 by dendritic cells is increased after local application of *Vitreoscilla filiformis* extracts on atopic dermatitis (Gueniche et al., 2008; Volz et al., 2014; Breton et al., 2017).

Oral probiotics could improve skin health by a gut-brain-skin (GBS) axis that reduces systemic and brain inflammation. The GBS axis improves nutrient absorption, which favors barrier synthesis (reviewed in Bowe and Logan, 2011). Oral ingestion of *Lactobacillus reuteri* diminishes perifollicular inflammation (Arck et al., 2010; Gueniche et al., 2014). Other probiotics targeting skin disorders improved atopic dermatitis, healing burns and scars, and even aging skin (Krutmann, 2012).

## Probiotics and Pharmacologic Therapies

The gut microbiota has an impact on drug absorption and hepatic metabolism and produces active metabolites that cannot be formed in the liver (Spanogiannopoulos et al., 2016). In addition, the response to or side effects of therapeutics could be influenced by some probiotics. B-glucuronidases produced by *Escherichia coli, Bacteroides vulgatus*, and *Clostridium ramosum* reactivate irinotecan from its inactive glucuronide form that is excreted via bile into the gastrointestinal tract in its toxic form, which is responsible for severe digestive toxicity (Wallace et al., 2010; Guthrie et al., 2017).

The gut microbiota affects the clinical response to anti-PD-1 immunotherapy in patients with advanced melanoma; a higher abundance of the *Ruminococcaceae* family and *Faecalibacterium* in fecal samples of patients was associated with longer progression-free survival (Gopalakrishnan et al., 2018). Dysbiosis induced by antibiotics was also associated with a poorer response to anti-PD-1 immunotherapy in cancer patients, while the abundance of *A. muciniphila* improved the therapeutic effect of these drugs (Routy et al., 2018). Fecal microbiota transplantation from cancer patients qualified as responders to anti-PD-1 immunotherapy into antibiotic-treated mice resulted in increased efficacy of immune therapy, with a positive correlation between these beneficial impacts and the abundance of *A. muciniphila* (Routy et al., 2018). In the same study, mice with a poor response despite fecal microbiota transplantation were supplemented with *A. muciniphila*, restoring the efficacy of anti-PD-1 therapy.

The *E. coli* strain Nissle 1917 increased amiodarone bioavailability in rats, while probiotic strains of *L. casei* slowed, although non-significantly, amiodarone absorption (Matuskova et al., 2014, 2017). Kim et al. showed that oral administration of *L. reuteri* K8 to mice reduced the area under the curve of acetaminophen compared with that of control mice (Kim et al., 2018).

## Conclusion

Modulating the microbiota by using probiotics or next-generation beneficial microbes constitutes a future perspective for the development of either nutritional or pharmaceutical tools to maintain health (Figure 1). Nevertheless, additional clinical research is needed to translate research into clinical practice, to refine the clinical indication of specific probiotic strains, to better understand the postbiotic effect of substances released by probiotic bacteria and the parabiotic effect of inactivated bacterial cell (Taverniti and Guglielmetti, 2011).

## Author Contributions

GW, LB, RE, J-MP, ID, PT, SL, and PC participate in writing specific paragraphs and re-reading the text.

### Conflict of Interest

PC is inventor listed on patent applications dealing with the use of *A. muciniphila* and its components to treat obesity and related disorders. PC is a cofounder of A-Mansia biotech SA. The remaining authors declare that the research was conducted in the absence of any commercial or financial relationships that could be construed as a potential conflict of interest.
